# Reading Visual Braille with a Retinal Prosthesis

**DOI:** 10.3389/fnins.2012.00168

**Published:** 2012-11-22

**Authors:** Thomas Z. Lauritzen, Jordan Harris, Saddek Mohand-Said, Jose A. Sahel, Jessy D. Dorn, Kelly McClure, Robert J. Greenberg

**Affiliations:** ^1^Second Sight Medical ProductsSylmar, CA, USA; ^2^Brigham Young University – IdahoRexburg, ID, USA; ^3^UMR-S 968, Institut de la VisionParis, France; ^4^CIC INSERM DHOS 503, National Ophthalmology HospitalParis, France

**Keywords:** retina, epiretinal prosthesis, sensory substitution, retinitis pigmentosa, blindness, perception, degeneration, sight restoration

## Abstract

Retinal prostheses, which restore partial vision to patients blinded by outer retinal degeneration, are currently in clinical trial. The Argus II retinal prosthesis system was recently awarded CE approval for commercial use in Europe. While retinal prosthesis users have achieved remarkable visual improvement to the point of reading letters and short sentences, the reading process is still fairly cumbersome. This study investigates the possibility of using an epiretinal prosthesis to stimulate visual braille as a sensory substitution for reading written letters and words. The Argus II retinal prosthesis system, used in this study, includes a 10 × 6 electrode array implanted epiretinally, a tiny video camera mounted on a pair of glasses, and a wearable computer that processes the video and determines the stimulation current of each electrode in real time. In the braille reading system, individual letters are created by a subset of dots from a 3 by 2 array of six dots. For the visual braille experiment, a grid of six electrodes was chosen out of the 10 × 6 Argus II array. Groups of these electrodes were then directly stimulated (bypassing the camera) to create visual percepts of individual braille letters. Experiments were performed in a single subject. Single letters were stimulated in an alternative forced choice (AFC) paradigm, and short 2–4-letter words were stimulated (one letter at a time) in an open-choice reading paradigm. The subject correctly identified 89% of single letters, 80% of 2-letter, 60% of 3-letter, and 70% of 4-letter words. This work suggests that text can successfully be stimulated and read as visual braille in retinal prosthesis patients.

## Introduction

Retinal prostheses restore partial vision to people blinded by outer retinal degenerative diseases such as Retinitis Pigmentosa (RP) or Macular Degeneration (Humayun et al., 2003). Recent results have demonstrated the ability of prosthesis users to read large letters and short words and sentences for some subjects (Sahel et al., 2011; Zrenner et al., 2011). But with the current spatial resolution of prosthetic vision, reading takes tens of seconds for single letters and minutes for short words, and requiring letters to be ∼1–20 cm high at normal (∼30 cm) reading distance (da Cruz et al., 2010; Sahel et al., 2011; Zrenner et al., 2011). While these results are in themselves are impressive, and the performance is expected to improve significantly with future prosthesis development, the practical application at current level is limited. For example, signs one might read while walking around have letters of a few centimeters in height, but are intended to be read from several meters distance, and it is not practical spending minutes to read each sign one might encounter.

An alternative is to use the prosthesis to create percepts in the form of braille letters (to be read visually rather than tactually). For example, letter recognition software could identify text (e.g., from a sign), which could then be translated into braille and stimulated via the visual prosthesis. This study addresses the feasibility of reading visual braille with retinal prostheses. The specific device used in this study is the Second Sight Argus^®^ II System (Second Sight Medical Products, Sylmar, CA, USA).

The Argus II System consists of a surgically implanted 60-channel stimulating microelectrode array, and inductive coil link used to transmit power and data to the internal portion of the implant, an external video processing unit (VPU), and a miniature camera mounted on a pair of glasses. The video camera captures a portion of the visual field and relays the information to the VPU. The VPU digitizes the signal in real time, applies a series of image processing filters, down-samples the image to a 6 by 10 pixilated grid, and creates a series of stimulus pulses customized to the individual user based on pixel gray-scale values. The Argus II System is commercially available in Europe (CE approval) and in clinical trial in the USA.

Here we present results showing that an Argus II subject can read visually stimulated braille. Performance is 89% correct for individual letters at 500 ms presentation, and 60–80% correct for short words, proving the feasibility of reading via visual braille.

## Materials and Methods

### Subject selection

Second Sight has 30 subjects enrolled in a clinical study, http://clinicaltrials.gov (NCT00407602). The subjects are blinded by the degenerative retinal disease Retinitis Pigmentosa (RP). RP causes the photoreceptor cells in the retina to die. Subjects are implanted with the Argus II retinal prosthesis system, which stimulates the surviving cells in the retina. Subjects have been implanted between 2 and 4.5 years. All subjects enrolled in the study have no cognitive impairments or learning ability deficiencies. A single subject was selected based on three criteria for this feasibility study: the ability to read (tactile) braille, spatial resolution high enough to isolate responses from six individual electrodes arranged in 3 by 2 pattern, and availability for testing. The subject is an experienced braille reader. The experiments were carried out September 2011 to March 2012 and approved by the Institutional Review Board at the location of the experiments (Centre Hospitalier National d’Ophtalmologie des Quinze-Vingts, Paris, France) and under the principles of the Declaration of Helsinki.

### Description of device

**Figure 1:**
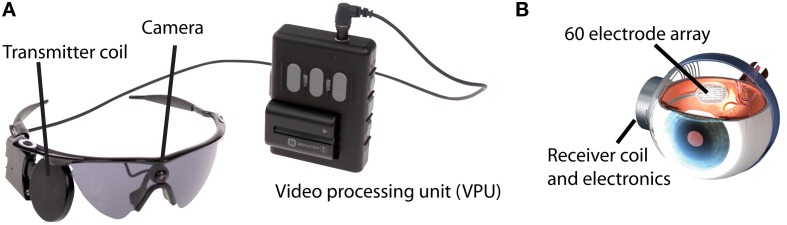
**Overview of Argus II system**. **(A)** External portion consisting of a miniature camera mounted on a pair of sunglasses, a Video Processing Unit (VPU), and a transmitter coil. **(B)** The internal portion, consisting of a receiver coil connected with a bus to a 60 electrode epiretinal array.

The Argus II System consists of an implantable device surgically implanted on and in the eye, and an external unit worn by the user. The external unit consists of a small camera and transmitter mounted on a pair of sunglasses and a VPU and battery that can be worn on a belt or shoulder strap (Figure 1A). The implanted portion (Figure 1B) consists of a receiving and transmitting coil and a hermetically sealed electronics case, fixed to the sclera outside of the eye, and an electrode array (a 6 by 10 array of 60 electrodes, 200 μm in diameter, 525 μm between nearest neighbor center to center cardinal axes) that is secured to the surface of the retina (epiretinally) inside the eye by a retinal tack. The electrode array is connected to the electronics by a metalized polymer cable that penetrates the sclera in the pars plana. The camera captures video and sends the information to the processor, which converts the image to electronic signals that are then sent to the transmitter on the glasses. The implanted receiver wirelessly receives these data and sends the signal to the electrode array via a small bus, where electric stimulation pulses are emitted. The controlled electrical stimulation of the retina induces cellular responses in retinal ganglion cells that travel through the optic nerve to the visual cortex and results in visual percepts.

### Selection of basis for stimuli

In this experiment, the Argus II System was used in “direct stimulation mode.” The camera was bypassed and individual electrodes were stimulated, controlled by a computer. Therefore, no visual reading software was used in these experiments.

**Figure 2:**
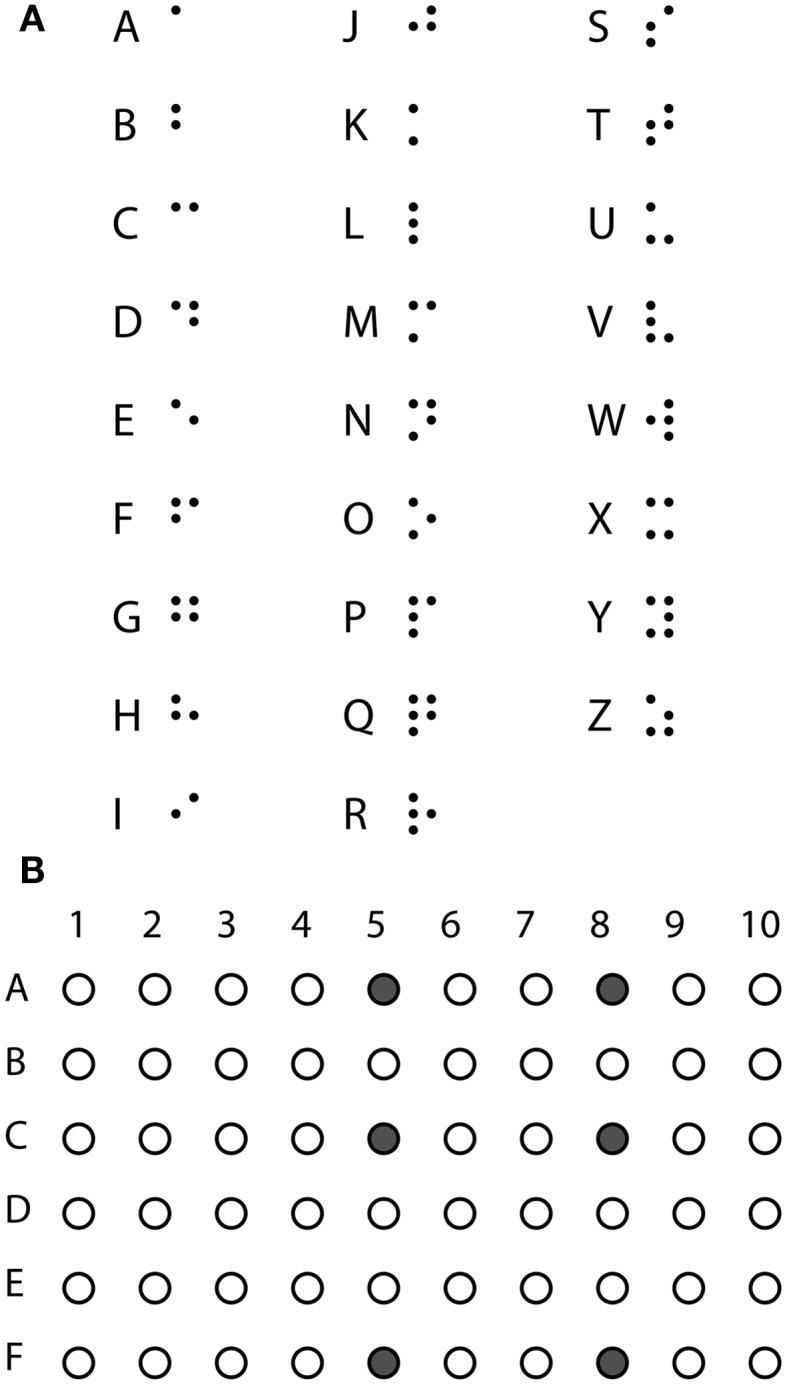
**Stimulating braille**. **(A)** The braille alphabet. **(B)** Six electrodes forming the basis of the braille stimulation used in the experiment.

The basis for the braille alphabet is a 3 by 2 array of dots, and each letter has a specific configuration (Figure 2A). For braille stimulation, sets of six electrodes were picked that spanned a 3 by 2 array. All six electrodes were stimulated at the same time with 20 Hz trains of 500 ms of 1 ms cathodic-anodic square pulses, i.e., 10 pulses. The current amplitude of pulses was set individually for each of the six electrodes to be 2.5–3 times the threshold for detection of a single electrode. A set of six electrodes resulting in a perceived stimulus of 3 by 2 dots was selected based on feedback from the subject (Figure 2B).

### Visual braille stimulation

The experimental paradigm was inspired by the character recognition experiments of the Argus II subjects (da Cruz et al., 2010; Sahel et al., 2011). For single letter recognition experiments, the 26 letters of the alphabet were split into three sets of 8 or 9 letters: set 1 (f, g, h, l, o, p, r, v), set 2 (a, c, d, i, k, m, s, w, y), and set 3 (b, e, j, n, q, t, u, x, z). The subject was aware of which letters were contained in the current set. Selection of the letters for each set was picked randomly with the one rule that letters with dots in the same geometric structure, but a single difference in distance would not be in the same set. Four such pairs exist, b-k, f-m, g-x, and h-u. For example, b and k are both made up of two dots in a vertical line with just a difference in spacing (see Figure 2A). With this rule, it would be possible to split the alphabet in first, middle, last thirds. But set 1 was picked as a pilot set of letters, avoiding the simplest letters made up of only one or two dots, and kept in the main experiment. Sets 2 and 3 were subsequently constructed of the remaining letters. The letters were then stimulated in random order with five repeats of each letter in an 8- or 9-alternative forced choice (AFC) paradigm. After each visual braille letter stimulation, the subject identified which letter was perceived, and the response was recorded by the experimenter. During the experiment, the subject could request that the letter set be repeated (i.e., he could be reminded of which letters were possible within the set). No other information was given to avoid biasing answers. A letter was presented as a 500 ms pulse train at 20 Hz with the subset of the six basis electrodes forming a given letter being active. To assure performance was not dependent on a narrow parameter range, the experiments were repeated with 40 and 60 Hz stimulation.

The subject was a native French speaker. To test the subject’s ability to read words in visual braille, the 10 most common 2-, 3-, and 4-letter words in French (Table 1) were picked based on usage frequency^1^ (New et al., 2001, 2004). Each word was presented with 500 ms per letter and 1000 ms break between letters. Considerations on the timing between letters are discussed in *Discussion*. The subject was informed that short words would be presented, but was not aware of which words were contained in the set. The order of the words was random and each word was stimulated once. The subject was allowed to request a single repetition of a word, but a guess would be considered a final answer. Responses were recorded by the experimenter.

**Table 1:** **List of words (in French)**.

2-Letter	3-Letter	4-Letter
de	les	dans
la	des	pour
et	que	elle
le	une	plus
il	est	mais
un	qui	nous
en	pas	avec
du	par	tout
je	sur	vous
ne	son	bien

### Analysis

Answers were summed and significance of the proportion of correct answers was determined based on binomial distributions (correct/wrong) and chance levels, 1/8 or 1/9 depending on letter set.

Error analysis was performed by comparing the braille pattern of the letter guessed by the subject to the pattern of the correct letter. The degree of error was determined by assigning one point for: each dot that was not perceived, each missing dot that was perceived (false positive), or each dot that was perceived in a wrong place and summing the points. This resulted in 0 degrees of error denoting a correct identification, and a maximum possible error of 6 degrees.

## Results

A subject, blinded by RP and implanted with the Argus^®^ II retinal prosthesis system, was presented visual braille via six electrodes arranged in a 3 by 2 pattern to span the braille alphabet. The subject had no cognitive or learning ability impairments, and was an experienced (tactile) braille reader.

### Single letter recognition

**Figure 3:**
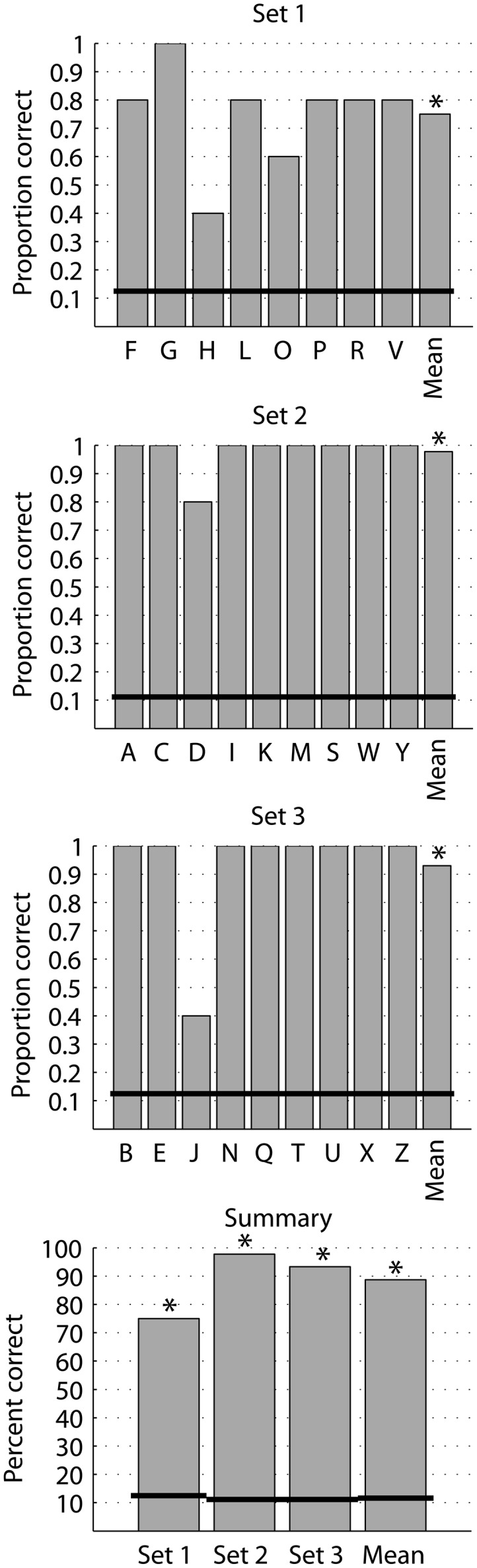
**Identification of single letters**. Proportion correct of identification of single letters in set 1 (8 AFC), set 2 (9 AFC), and set 3 (9 AFC) and the summary percent correct. Each letter was presented five times in random order within its set. The black horizontal lines denote chance level for the respective set. *p < 0.001 (binomial probability distribution).

Single letters were stimulated in sets of 8 or 9 letters in an AFC paradigm with five repetitions of each letter. Single letters were presented for 500 ms. Letter recognition was high for all presented letters. The detection rate at 20 Hz stimulation for the three letter sets ranged between 75 and 98% with a mean of 89% correct, and all were highly significantly above chance level (*p* < 0.001; Figure 3). Stimulation at 40 and 60 Hz yielded 85 and 77% mean correct, both significantly above chance recognition (*p* < 0.001) and not significantly different from the recognition rate at 20 Hz stimuli (data not shown).

**Figure 4:**
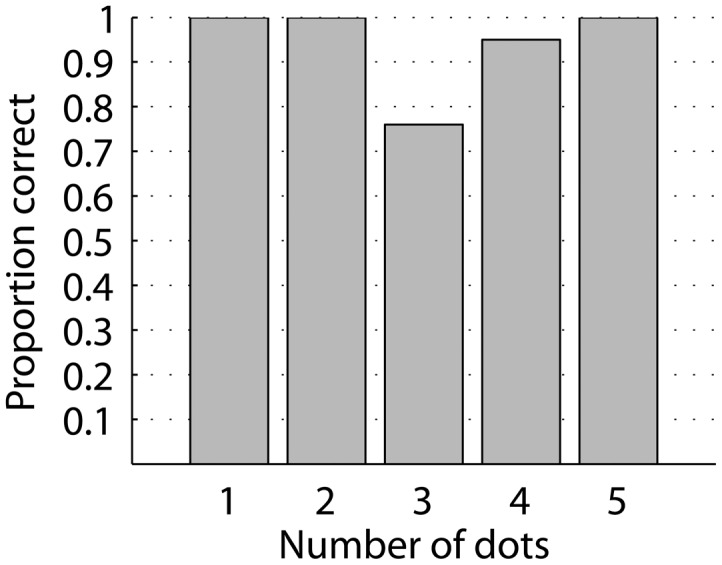
**Identification of single letters as a function of letter complexity, measured as the number of dots forming a letter**. All letter complexities have high identification rate and there is no systematic change in identification with complexity.

While the complexity of letters varies, there is no indication that performance depended on the complexity of letters, measured as the number of dots in a letter (Figure 4).

**Figure 5:**
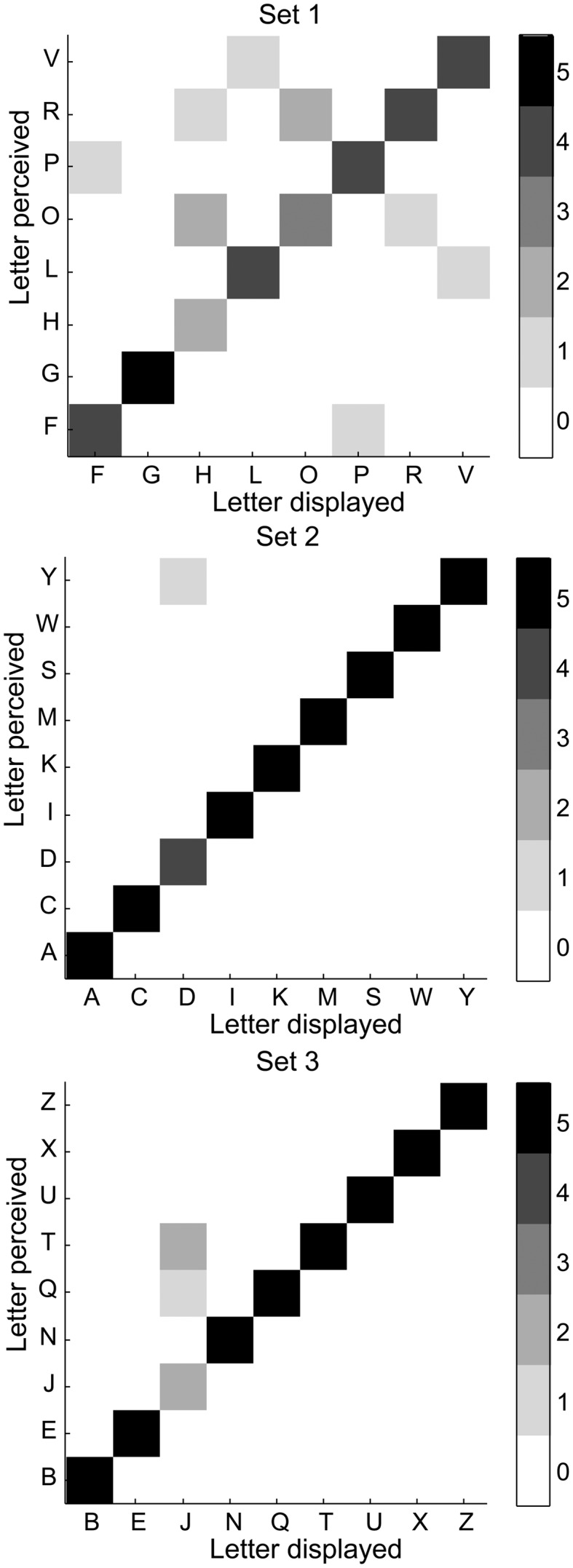
**Error matrices**. Matrix plots of letter perceived (*y*-axis) as a function of the letter displayed (*x*-axis). The ideal case would be a diagonal matrix.

**Figure 6:**
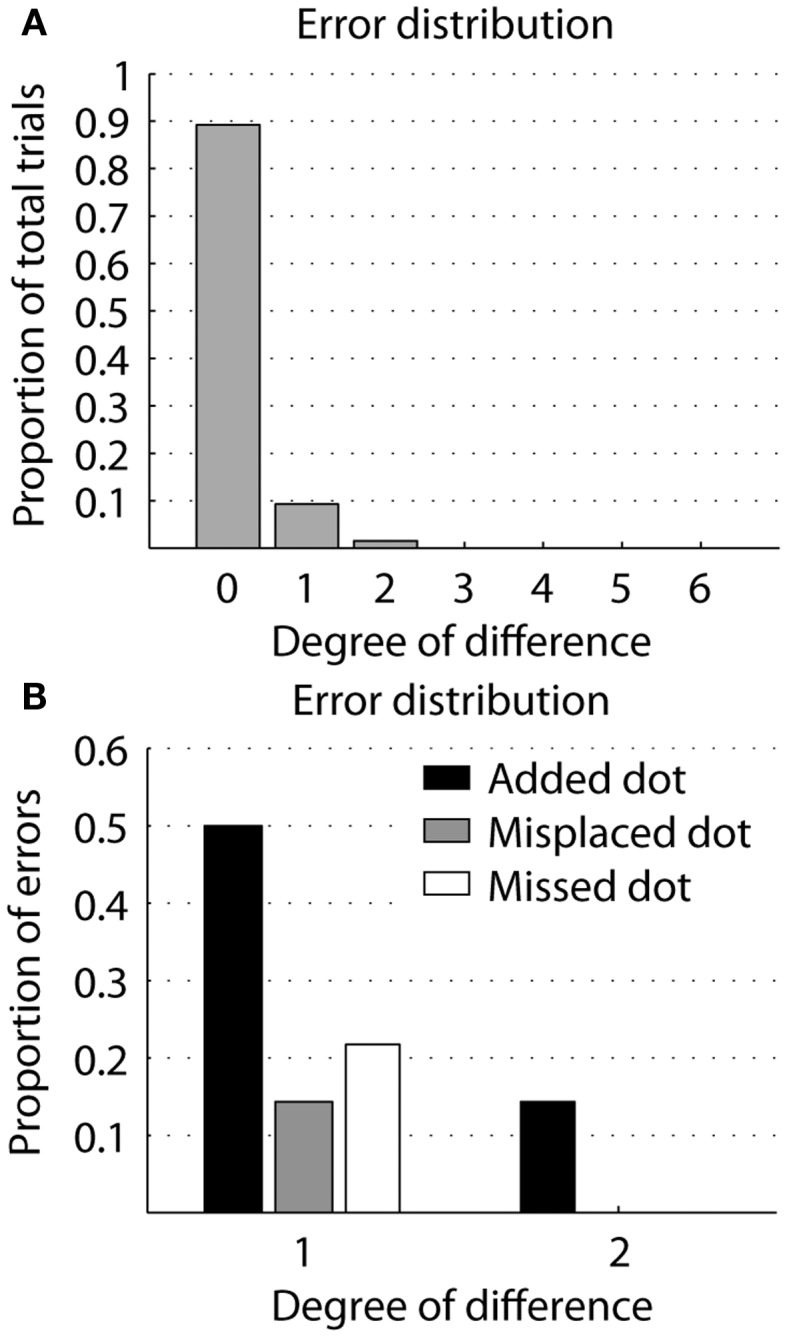
**Degree of error in recognizing single letters**. **(A)** The distance in error between the stimulated and perceived letter. Each degree is a perceived dot added, missed, or perceived in a wrong location. Zero degree difference is correct identification. Most errors are a single degree. Theoretically, the maximum error is 6°. **(B)** The type of error, an extra perceived dot, a misplaced dot, or a missed dot, as a function of the distance in error.

Error matrices show the perceived letter as a function of the displayed letter (Figure 5). There is no systematic error in misperceived letters. To determine a degree of error, the perception errors were scored the perception by adding a point for each extra perceived dot, missed dot, or dot perceived in a wrong location. Zero degree error is a correct perception (89%) and the maximum possible number of errors with a 6-dot basis is 6 degrees of error. Nine percent of the perceptions had 1 degree (82% of all errors), 2% had 2 degrees of error (18% of all errors), and there were no higher errors (Figure 6A). Splitting the errors up in extra perceived, missed, or dot in wrong location, we see that by far the most errors (64%) are caused by one or two extra perceived dots, while 21 and 14% respectively are caused by a missed dot or a dot perceived in the wrong location (Figure 6B). Further, the error matrices (Figure 5) show that electrode F5, representing the lower left dot is involved in 9 of the total of 14 errors.

### Word recognition

**Figure 7:**
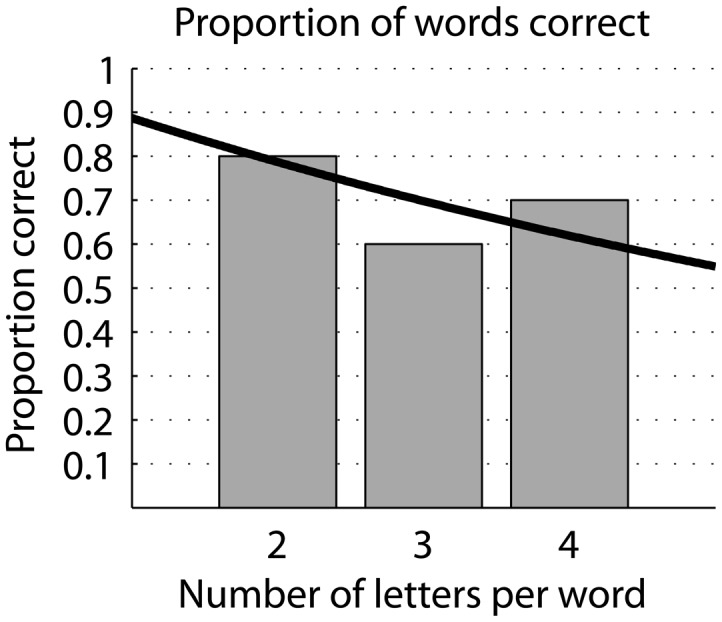
**Recognition of braille words**. Proportion correct identification of 2-, 3-, and 4-letter words. Black line represents the expected proportion correct given a proportion of single letter identification rate of 89%.

The subject was presented 10 2-, 3-, and 4-letter words (Table 1) and correctly identified eight, six, and seven words respectively (Figure 7). The proportion of word recognition was highly significant based on random letter presentation (For example, since the whole alphabet was available, chance of a 2-letter word is 1/26^2^ = 0.0015). The proportion of word recognition is not significantly different from what would be predicted by the single letter recognition proportion [0.89^(word length)^; Figure 7]. Eighty-nine percent is the average proportion correct from eight and nine AFC experiments. It is reasonable to expect the number is similar in a 26 AFC task (ignoring the use-frequency of individual letters in regular text). Comparing the presented and guessed words, the nine word errors contained a total of 15 single letter errors. Of these, eight were single dot errors, five were a missed letter, and one involved flipping the order of two letters (counts as two single letter errors). Only one error contained multiple dot (three) errors.

## Discussion

This work shows that an Argus II user can read both single letters and short words in visually stimulated braille. The subject recognized 89% of presented letters. Eighty-two percent of errors were due to a single dot misperception, and there is no indication that the complexity of the letter played a role in perception. Sixty-four percent of the errors were caused by the perception of an extra dot. Similarly, the electrode representing the lower left dot (electrode F5) was involved in 64% of all the errors, including 6 of the total 11 extra perceived dots. This indicates that improving the performance of that electrode will significantly improve the results. The subject also identified eight of 2-, six of 3-, and seven of 4-letter words of a total of 10 presented words of each length. It is reasonable to expect the performance will improve with training. The subject is an experienced braille reader. While we did not test it specifically in this study, it is safe to assume a 100% identification rate for tactile braille. Thus the discrepancy is due to visual stimulus comprehension and not braille comprehension. This opens the possibility for the Argus II users to read text by making a sensory substitution to visual braille.

### Comparison to other visual prosthetic stimulation

Dobelle et al. (1976) stimulated visual braille with a visual cortex prosthesis. Presenting randomized single letters presented for 500 ms to a subject, they reported 73–85% correct responses, depending on the exact experimental paradigm. These results are similar to the results presented here. Dobelle et al. (1976) picked six electrodes spanning a 3 by 2 array of perceived phosphenes. Interestingly, the perceived locations of the six electrodes were “scrambled” compared to their array locations. We expect these non-linear discrepancies from the retinotopic map are due to their large electrode array (several cm) covering several sulci and gyri. The phosphene locations of the electrodes in the Argus II subject were more linearly arranged one-to-one, as expected when stimulating the retinotopic space of the retina.

Other retinal prostheses have the ability to function in a “direct stimulation” mode (Wilke et al., 2011a; Zrenner et al., 2011). To the best of our knowledge, these groups have not experimented with visual braille in direct stimulation. Real world use of visual braille for reading requires visual processing filters, such as character recognition software, to allow for translating text into braille. Thus the Argus II system is the only currently available system able to apply visual processing filters, such as character recognition software, to allow for translating text into braille for stimulation in real world use.

### Considerations on braille reading speed

The stimulation time used in these experiments (500 ms per letter and 1000 ms between letters) is significantly faster than the current reading speed reported with retinal prostheses (tens of seconds per letter; da Cruz et al., 2010; Sahel et al., 2011; Zrenner et al., 2011). The current study did not explore details on how stimulation time affects perception. In a short pilot experiment, we did set the stimulation time to 250 ms in a run of letter set 1, and found that the subject perceived 77.5% of the letters correctly. This is not significantly different from the 75% correct at 500 ms (Figure 3). This indicates that it is possible to perceive visual braille at very short presentation times of down to, at least, 250 ms.

While shortening the presentation time of individual letters may increase word reading speed, we expect a limiting factor is the timing between letters and words. Recent experiments with direct stimulation in retinal prostheses indicate that the persistence of a phosphene is 150–200 ms (Lauritzen et al., 2011; Wilke et al., 2011b). Similarly, Dobelle et al. (1976) reported that “at frames faster than 4s^−1^, presentations tend to blur” indicating that phosphenes generated by direct cortical stimulation have a similar persistence. These findings indicate that a theoretical lower limit for the interval for visual braille reading is slightly higher than 150–200 ms, say ∼250 ms. If letter (and word-space) presentations are also ∼250 ms, i.e., ∼500 ms per letter plus space, a realistic goal for reading speed is ∼120 letters per minute. This is an adequate speed for reading signs and shorter messages.

### Considerations on braille reading performance

In this experiment, single letter performance was 89% correct, and performance of reading of short words aligned well with expectation based on single letter performance (Figure 7). While the single letter performance is high, and we expect it to get better with training, a simple multiplication of probabilities would result in a larger amount of errors for just slightly longer words. But this is alleviated by the increased structure of longer words and context of sentences (e.g., Baayen et al., 1995; New et al., 2004). For example, missing a letter in the word “restaurant” does not alter it to something unrecognizable.

### Considerations for prosthetic applications

Implementing a visual braille function in prosthetic vision requires implementing optical character recognition software for reading text in the VPU. Such software is common use (e.g., Google Goggles)^2^ and Open Source codes are available^3^. Reading identified text is only part of the problem. Identifying text in the environment is the other. Different groups have published algorithms for detecting and reading text in natural scenes (Chen and Yuille, 2004; Shen and Coughlan, 2012). In particular, Chen and Yuille (2004) report a success rate of detecting and reading text of more than 90% (detecting 97.2% of all text in natural images, and reading 93% of the detected text). Algorithms like this are only expected to improve in the future.

Further, the user would need to read visual braille. The subject in this study reads braille, but only about 10% of blind people read braille^4^. Interestingly, the subject in the Dobelle et al. (1976) study did not know (tactile) braille at the onset of the study. During the study, they tested both tactile and visual braille, and the subject averaged only 28% correct letter identification using tactile braille as opposed to 73–85% letter identification using visual braille. This validates the notion that visual braille is a different modality than tactile braille. While knowledge of tactile braille is useful, it is likely not a necessity for succeeding in reading visual braille.

## Conclusion

In summary, stimulation of visual braille is feasible for conveying text to visual prosthesis users, and the technology needed can readily be implemented. It is a requirement that the user is able to read braille, but this can be learned with limited effort if the user does not already have this ability.

## Conflict of Interest Statement

Thomas Z. Lauritzen, Jessy D. Dorn, Kelly McClure, Robert J. Greenberg are employees of and have financial interests in Second Sight Medical Products. Jordan Harris has no conflicts of interest.

## Footnotes

^1^ www.lexique.org

^2^ http://www.google.com/mobile/goggles

^3^ http://code.google.com/p/tesseract-ocr/

^4^ http://nfb.org/braille-campaign
